# Machine Learning for Seed Quality Classification: An Advanced Approach Using Merger Data from FT-NIR Spectroscopy and X-ray Imaging

**DOI:** 10.3390/s20154319

**Published:** 2020-08-03

**Authors:** André Dantas de Medeiros, Laércio Junio da Silva, João Paulo Oliveira Ribeiro, Kamylla Calzolari Ferreira, Jorge Tadeu Fim Rosas, Abraão Almeida Santos, Clíssia Barboza da Silva

**Affiliations:** 1 Agronomy Department, Federal University of Viçosa, Viçosa MG 36570-900, Brazil; laercio.silva@ufv.br (L.J.d.S.); joaop.ribeiro@ufv.br (J.P.O.R.); abraaoufs@gmail.com (A.A.S.); 2 Chemistry Department, Federal University of Viçosa, Viçosa MG 36570-900, Brazil; kamylla.ferreira@ufv.br; 3 Soil Science Department, University of São Paulo, Piracicaba SP 13418-260, Brazil; jorge.fimrosas@usp.br; 4 Entomology Department, Federal University of Viçosa, Viçosa MG 36570-900, Brazil; 5 Laboratory of Radiobiology and Environment, University of São Paulo-Center for Nuclear Energy in Agriculture, 303 Centenário Avenue, Piracicaba SP 13416-000, Brazil; clissia_usp@hotmail.com

**Keywords:** germination prediction, linear discriminant analysis, Fourier transform near-infrared spectroscopy, radiographic images, *Urochloa brizantha*

## Abstract

Optical sensors combined with machine learning algorithms have led to significant advances in seed science. These advances have facilitated the development of robust approaches, providing decision-making support in the seed industry related to the marketing of seed lots. In this study, a novel approach for seed quality classification is presented. We developed classifier models using Fourier transform near-infrared (FT-NIR) spectroscopy and X-ray imaging techniques to predict seed germination and vigor. A forage grass (*Urochloa brizantha*) was used as a model species. FT-NIR spectroscopy data and radiographic images were obtained from individual seeds, and the models were created based on the following algorithms: linear discriminant analysis (LDA), partial least squares discriminant analysis (PLS-DA), random forest (RF), naive Bayes (NB), and support vector machine with radial basis (SVM-*r*) kernel. In the germination prediction, the models individually reached an accuracy of 82% using FT-NIR data, and 90% using X-ray data. For seed vigor, the models achieved 61% and 68% accuracy using FT-NIR and X-ray data, respectively. Combining the FT-NIR and X-ray data, the performance of the classification model reached an accuracy of 85% to predict germination, and 62% for seed vigor. Overall, the models developed using both NIR spectra and X-ray imaging data in machine learning algorithms are efficient in quickly, non-destructively, and accurately identifying the capacity of seed to germinate. The use of X-ray data and the LDA algorithm showed great potential to be used as a viable alternative to assist in the quality classification of *U. brizantha* seeds.

## 1. Introduction

Seed quality is an important factor in agricultural production, with a direct impact on yield [1]. In plant breeding, the use of high-quality seeds reduces costs of field experiments and increases the probability to identify a better crop variety. In the seed industry, quality assurance programs rely on numerous methods to certify seed quality attributes, such as germination and vigor tests [2]. These procedures have limitations related to time consumption, subjectivity, and the destructive nature of assessing seed quality [3,4,5]. In fact, there is a growing demand for efficient methods that can provide a quick, reliable, non-destructive, and objective detection of seed quality [6].

Usually, changes in chemical composition and internal anatomical characteristics of seeds are correlated with loss of viability and vigor [5], but these changes are unlikely to be identified by visual inspection. Meanwhile, methods based on spectrometry and X-ray imaging techniques have been successfully used to collect data on complex traits related to seed quality. For instance, Fourier transform near-infrared (FT-NIR) spectroscopy has proved great potential in detecting seed compounds by acquisition of a large number of spectral details [6,7,8,9,10,11,12]. FT-NIR spectroscopy is based on the absorption of electromagnetic radiation at wavelengths ranging from 780 to 2500 nm [13]. Therefore, it offers versatility for direct and simultaneous measurements of several constituents in seed samples [10,14,15,16,17]. On the other hand, X-ray imaging is based on differences in X-ray attenuation in different types of tissues [18]. Hence, it can reveal the physical state of the seed, i.e., its internal morphology [5]. Although these techniques have high potential for seed quality classification, combining datasets may generate new information about seed samples or improve the performance of classifiers [19].

Recent advances of machine learning algorithms have revolutionized the agricultural industry because they are the basis for building models to classify products, particularly quality attributes of seeds. Robust algorithms can capture linear and non-linear relationships, and they can achieve high classification accuracy. Several algorithms have been proven to be effective for solving problems in many fields of research, such as linear discriminant analysis (LDA), partial least squares discriminant analysis (PLS-DA), random forest (RF), naive Bayes (NB), support vector machine with linear (SVM-*l*) and radial basis (SVM-*r*) kernel, and artificial neural network (ANN) [6,9,11,19,20]. However, different algorithms operate differently and they can have different performances [20].

Although optical-based methods can generate accurate information on seed quality, merging datasets through machine learning algorithms may further improve classification performance. To the best of our knowledge, there have been no attempts in using FT-NIR combined with X-ray image data to classify seed quality. Therefore, using seeds of *U. brizantha* grass as a model, we tested whether merged data from FT-NIR and X-ray imaging with machine learning algorithms can improve the predictions of seed germination and vigor.

## 2. Materials and Methods

### 2.1. Plant Material

In this study, we investigated 200 seeds of *U. brizantha* (MG13 Braúna cultivar) produced in the crop season of 2019/2020. Each seed was identified, and then spectral data and radiographic images were obtained individually, followed by evaluation of seed germination and vigor. Each seed was numbered and classified according to its physiological potential.

### 2.2. NIR Data Collection and Preprocessing

The absorbance data from each seed was measured using a Thermo Scientific Antaris II FT-NIR spectrometer with a spectral range from 1000 to 2500 nm, with each spectrum represented by an average of 32 scans measured with an 8 cm^−1^ resolution, resulting in 3112 channels. This instrument operates in an integrating sphere with a diffuse reflectance module and spectra were obtained in reflectance mode as log (1/R). It took approximately 30 s to obtain the spectrum of each seed.

We preprocessed the spectral data by removing the external noises using the “prospectr” package [21] in the R software [22]. A signal pretreatment was performed using autoscaling and first-order derivative transformation with Savitzky–Golay smoothing, followed by data binning (window = 10), which reduced the number of spectral bands from 3112 to 310. Data binning is a form of quantization of the spectral data, in which the original values are replaced by single central values to reduce the effect of noise during the spectrum acquisition [20].

### 2.3. X-Ray Imaging

Initially, the seeds were fixed on an adhesive paper in groups of 50 seeds. Then, radiographic images were generated using a Faxitron MX-20 device (Faxitron X-ray Corp, Wheeling, IL, USA). Seeds were exposed to radiation for 10 s with a voltage adjustment of 23 kV at a focal length of 41.6 cm. The contrast of the X-ray images was adjusted to optimize the visualization of internal seed tissues, and the images were saved in tagged image file format (TIFF).

We used the IJCropSeed tool to analyze the X-ray images [23]. This tool provided 17 descriptors related to the following seed morphometric characteristics: area, perimeter, circularity, width, height, feret, aspect ratio, roundness, solidity, relative density, integrated density, median gray level, skewness, kurtosis, internal free space, and seed filling (filling.1 and filling). The details on the descriptors can be found in Medeiros et al. [23].

### 2.4. Physiological Analysis

The seed germination rate and the time required to produce normal seedlings were evaluated. A normal seedling showed vigorous growth without any visible defects. The experiment was conducted using transparent plastic boxes (11.0 × 11.0 × 3.5 cm^3^) with two blotting papers moistened with distilled water (1:2.5, g mL) placed inside the boxes [24]. The boxes were kept at daily temperature alternations (16 h at 15 °C and 8 h at 35 °C), and seed germination (root protrusion) was evaluated daily until 21 days after sowing.

### 2.5. Machine Learning for Seed Quality Classification

#### 2.5.1. Germination and Vigor Classes

Seeds were classified according to germination capacity (root protrusion at 21 days) and their vigor. Seed vigor was calculated based on germination speed (time required to generate a normal seedling). Subsequently, three seed classes were created: Class 1: non-germinated seeds; Class 2: rapid germination—normal seedlings produced within 9 days; Class 3: slow germination—normal seedlings produced later than 9 days. The period of 9 days was defined based on the accumulated germination curve, with approximately 71% of germinated seeds.

#### 2.5.2. Machine Learning Methods

The FT-NIR and X-ray data were organized into two datasets and analyzed individually. Later, the variables of each technique were used to create another dataset combining information from the two techniques. Each dataset was arranged in an X matrix (predictors) and data from the germination test (seed classes) were arranged in the Y vector (response). The predictive models were created using five machine learning algorithms: LDA, PLS-DA, RF, NB, and SVM-*r*. Data analysis was performed by R software using the “caret” package [25]. The “caret” package was also used to calculate the most important predictor variables for the models, in which the variable importance was dimensioned to a maximum value of 100 and a minimum value of 0. The hyperparameters used in each model are shown in Table 1.

#### 2.5.3. Model Validation

The training set comprised 60% of the data, and the remaining 40% were used to test the models. The model performance was evaluated through cross-validation (fold = 5), and the quality of predictions was measured based on overall accuracy, sensitivity, and specificity metrics obtained by the “caret” package.

## 3. Results

### 3.1. Spectral Overview and Internal Seed Morphology

**Figure 1:**
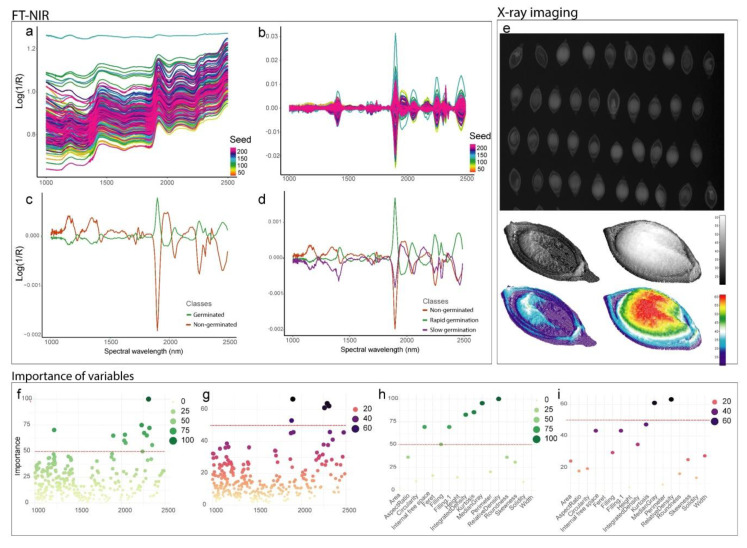
Quality classification of *U. brizantha* seeds based on Fourier transform near-infrared (FT-NIR) spectroscopy and X-ray imaging: (**a**) raw NIR spectra; (**b**) spectra preprocessed to establish predictive bands and reduce the effect of noise; (**c**) mean spectra for classes of germination capacity and (**d**) seed vigor; (**e**) X-ray images and 3D projections of seeds based on grayscale; (**f**) importance of wavelengths in FT-NIR based on the PLS-DA model for classes of germination capacity and (**g**) seed vigor; (**h**) importance of X-ray image descriptors based on the LDA model for classes of germination capacity; and (**i**) PLS-DA model for classes of seed vigor.

The raw NIR spectra are shown in Figure 1a. Since raw spectral data may present noise and compromise the analysis, data were preprocessed using autoscaling, first-order derivative transformation with Savitzky–Golay smoothing and data binning (Figure 1b). This allowed for reducing the number of wavelengths from 3112 to 310, and establish predictive wavelengths to create the machine learning models for training. The mean spectra showed differences between classes of germination capacity for most bands, with alternating absorbance peaks between classes depending on the NIR region (Figure 1c), and a similar behavior was also shown between classes of vigor (Figure 1d).

X-ray images of individual seeds were used to assess morphometric descriptors, including tissue integrity features (Figure 1e). Healthy seeds and seeds with embryonic malformation, mechanical damage, and deteriorated tissues were identified based on the grayscale values of the pixels in the images, which were directly associated with seed quality traits. In the colormap, hot and cold colors indicate high and low grayscale values, respectively (Figure 1e). Regions with higher grayscale values in the image represent lower penetration of the X-ray, which is directly associated with higher tissue density. Soft tissues, such as damaged tissues, show higher absorption of the X-ray beam as it passes through the tissue.

In this study, the importance of variables used to develop the models was calculated. Variable importance represents the statistical significance of each variable in the data set concerning its effect on the model generated [26]. For germination capacity, the PLS-DA (Figure 1f) and the LDA (Figure 1h) model revealed eleven wavelengths (1221, 1902, 2029, 2037, 2045, 2230, 2259, 2289, 2309, 2320, and 2351 nm) and six variables from X-ray images (relative density, integrated density, median gray level, kurtosis, filling.1, and internal free space), respectively, with greater contribution for the models (threshold >50%). For seed vigor, five wavelengths contributed more to the PLS-DA model (1889, 1902, 2289, 2309, 2259 nm) (Figure 1h), and two X-ray variables (relative density and median gray) reached a contribution greater than 50% (Figure 1i).

### 3.2. Machine Learning Models

We developed models for seed quality classification using X-ray image features and NIR data, individually or combined. The models were developed to predict seed germination capacity and seed vigor (speed to generate normal seedlings).

### 3.3. Germinated and Non-Germinated Seed Classification

The classes of germination capacity were unbalanced with 147 of germinated seeds and 58 of non-germinated seeds. In the testing set, the PLS-DA algorithm showed better performance for FT-NIR spectroscopy data, with 82% accuracy (Table 2). This result indicates a great potential of the FT-NIR spectroscopy technique for classifying seed germination capacity. The model using X-ray data individually achieved high accuracy, from 84 to 90%, depending on the algorithm. Combining FT-NIR and X-ray data, the performance of the models improved in relation to the classification using only FT-NIR data; however, it remained the same or less compared to the classification using only X-ray features. The RF model was highlighted with the merged data, with an accuracy of 85%. Overall, sensitivities below 74% and specificities above 82% were obtained.

### 3.4. Seed Vigor Classification

Predictions for seed vigor reached lower accuracy (43–68%) for all algorithms (Table 3). The classifiers developed using the FT-NIR data individually achieved lower accuracy (<61%) with lower sensitivity (<55%) and specificity (<79%). On the other hand, the model showed better performance with the X-ray data, reaching 68% accuracy in the test set via PLS-DA, and 64% accuracy in the cross-validation via SVM-*r*. FT-NIR combined with X-ray data had an intermediate performance for seed vigor classification. The best result was obtained with the RF algorithm (59% and 62% accuracy, for cross-validation and testing, respectively).

## 4. Discussion

The use of optical sensors to identify spectral and physical properties of seeds has contributed to quickly, accurately, and non-destructively obtaining valuable chemical and structural information related to seed performance. Although this technology is effective in solving problems in many fields, there is still a demand for non-destructive, fast, accurate, and online predictive methods for assessing seed quality by the seed industry [2]. In this study, we presented a new methodology based on merged data to predict germination capacity and seed vigor using FT-NIR and X-ray images, which was validated using seeds of *U. brizantha*.

Our results showed high accuracy of the models developed to classify seeds according to their germination capacity, and moderate performance for predicting seed vigor. The models developed using X-ray data achieved the highest precision, with an accuracy of 0.90 for germination and 0.68 for vigor prediction via the LDA and PLS-DA algorithms, respectively. The variables related to tissue density had the most contribution to seed quality classification. This fact reveals the direct relationship between physical characteristics of seeds (tissue integrity) and their physiological quality.

Differences in tissue densities in radiographic images are associated with morphological alterations and anatomical properties [6]. The relationship between tissue density parameters and seed germination was also reported for other species, indicating that X-ray imaging has great potential to be employed for seed quality classification [7,27,28]. Nevertheless, since X-ray images only show the physical state of an object, caution is necessary when using this technique, as seeds are living and complex organisms influenced by many factors [5]. Consequently, this relationship may not always be detected using only the X-ray technique [6].

The use of NIR spectroscopy methods combined with X-ray imaging can provide both seed chemical composition and physical integrity measurements [10,14,15,16,17]. The NIR spectra comprise bands of higher wavelengths arising from overlapping absorptions corresponding to chemistry bond combinations such as C-H, O-H, and N-H [3]. The spectral bands that had greater contribution to classify the germination capacity of *U. brizantha* seeds were 1221, 1902, 2029, 2037, 2045, 2230, 2259, 2289, 2309, 2320, 2351 nm. The chemical compounds related to these wavelengths are amino acids, carbohydrates (cellulose, hemicellulose, pectic polysaccharides, pyranose compounds, starch, and sucrose) and nucleic acids [11,13,25,29,30]. Using the NIR data, the best algorithm to classify the seed germination capacity was PLS-DA, reaching an accuracy of 82%. In previous studies, the use of FT-NIR showed higher accuracy (90–100%) for seed viability classification using the PLS-DA algorithm [9,10,12]. However, it is important to mention that this is a pioneering study with the application of NIR for *U. brizantha* seed analysis. Therefore, further research is needed to elucidate the lower performance achieved, which may be related to the seed characteristics (e.g., presence of palea and lemma, greater amount of dead tissue—endosperm, and reduced embryo size).

The use and combination of different techniques have gradually increased in seed technology, especially for detecting seed viability [12,31,32,33,34,35]. Combinations based on merged data have shown the potential to increase reliability on seed classification when compared to the use of individual analytical techniques [3,19]. In the present study, we combined information from both FT-NIR and X-ray techniques into five machine learning models in order to find the best classifier. The model achieved an accuracy of 85% with the RF algorithm to predict the seed germination capacity. In total, 310 variables from FT-NIR, and 17 variables from X-ray imaging were used. Conversely, we did not have similar results for seed vigor prediction, where the models showed less accuracy (<62%).

**Figure 2:**
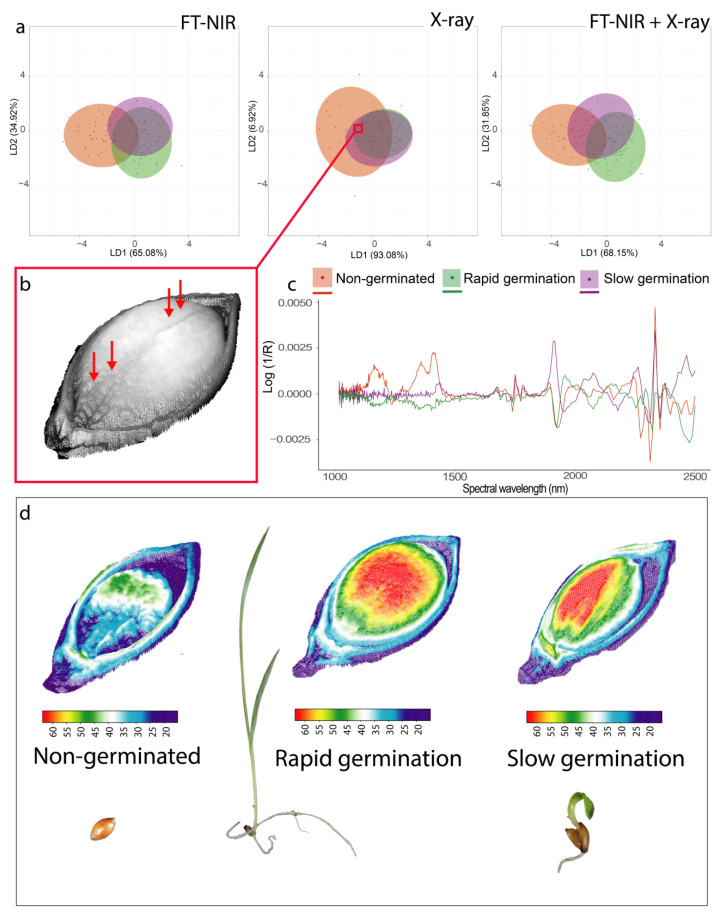
(**a**) Score plots of the linear discriminant analysis (LDA) for quality classification of *U. brizantha* seeds based on germination speed using Fourier transform near-infrared (FT-NIR) spectroscopy, X-ray imaging, and the combination of FT-NIR spectroscopy and X-ray imaging. (**b**) Radiographic image of one seed showing mechanical damages (red arrows). (**c**) Characterization of spectral absorbance signature of seeds with different vigor level. (**d**) 3D projections of X-ray images based on grayscale for classes of non-germinated seed, rapid and slow germination speed.

The lower accuracy of the models developed for seed vigor classification may be associated with the complexity of seed behavior, which is also influenced by environmental conditions [1]. For instance, our model distinguished class 2 (rapid germination) from class 1 (non-germinated seeds), but not from class 3 (slow germination) (Figure 2a). Interestingly, seeds with soft mechanical damages in the embryonic axis (Figure 2b) were not identified by the model as non-germinated seeds (class 1); instead, they were erroneously classified as class 2 or 3.

We selected three seeds of each class and presented their spectra and 3D projection of X-ray images based on grayscale to simplify the relationship between the methods tested in this paper. We observed differences among classes for absorbance values across the spectrum (Figure 2c) and grayscale values in the X-ray images (Figure 2d), which directly affected seed performance (Figure 2d).

Our findings showed that the model built with the X-ray dataset has higher accuracy for seed quality classification using the LDA algorithm. Additionally, when X-ray data were combined with FT-NIR data, the model also showed high performance. These results point out new perspectives to combine two sensors as a powerful tool for predicting seed quality, e.g., while FT-NIR can provide information on chemical composition, the X-ray images give information on the tissue integrity. However, despite this combination being an interesting approach, the results obtained with this work highlighted the X-ray technique as the most reasonable option to analyze the seeds, considering the high precision achieved in the models. Although our models are specific for *U. brizantha* seeds, the methodology proposed can be widely applied for other species. To the best of our knowledge, this is the first attempt to combine FT-NIR and X-ray imaging data to predict seed quality using machine learning models, and our findings can be a guide for the development of in-depth studies.

## 5. Conclusions

This study investigated the combination of FT-NIR spectroscopy and X-ray imaging to predict seed quality traits (germination and vigor). The proposed approach is sensitive to obtain information on the capacity of seeds to germinate (85% accuracy). On the other hand, it was not possible to reliably estimate the seed vigor (62% accuracy). The method can be an alternative to rapid, non-destructive, and accurate classification of seed quality by merging FT-NIR spectroscopy and X-ray imaging data. Regarding the application of the techniques individually, the X-ray approach was highlighted as the most viable option, as it does not need data preprocessing and deals with fewer variables than FT-NIR, resulting in less time and complexity for this analysis.

## Figures and Tables

**Table 1:** Hyperparameters used in the machine learning models.

Algorithm	Hyperparameters	FT-NIR	X-Ray Imaging	FT-NIR + X-Ray Imaging
		Values		
		Classification of seed germination		
LDA	dimensions	1	1	1
PLS-DA	components	6	1	3
RF	trees	36	15	290
NB	Laplace correction, Kernel, adjust	0, TRUE, 1	0, FALSE, 1	0, FALSE, 1
SVM-*r*	Sigma, cost	0.003315536, 4	0.05969127, 0.5	0.003371439, 2
		Classification of seed vigor		
LDA	dimensions	2	2	2
PLS-DA	components	6	3	6
RF	trees	275	2	290
NB	Laplace correction, Kernel, adjust	0, TRUE, 1	0, TRUE, 1	0, TRUE, 1
SVM-*r*	Sigma, cost	0.002813337, 2	0.07259337, 0.25	0.002386695, 2

Note: LDA—linear discriminant analysis; PLS-DA—partial least squares discriminant analysis; RF—random forest; NB—naive Bayes; SVM-*r*—support vector machine with radial basis kernel.

**Table 2:** Number of seeds correctly classified for germination capacity using descriptors generated by Fourier transform near-infrared (FT-NIR) spectroscopy, X-ray imaging, and the combination of FT-NIR spectroscopy and X-ray imaging.

Method	Feature	FT-NIR		X-Ray Imaging		FT-NIR + X-Ray Imaging	
		Cross-Validation	Testing	Cross-Validation	Testing	Cross-Validation	Testing
		(n = 121)	(n = 79)	(n = 121)	(n = 79)	(n = 121)	(n = 79)
		Hits (Total)		Hits (Total)		Hits (Total)	
LDA	Germinated	-	47(56)	-	54(56)	-	47(56)
	Non-germinated	-	17(23)	-	17(23)	-	14(23)
	Accuracy	0.68 ± 0.11	0.81	0.85 ± 0.07	**0.90**	0.74 ± 0.09	0.77
	Sensitivity	0.47 ± 0.16	0.74	0.63 ± 0.14	0.74	0.58 ± 0.09	0.61
	Specificity	0.78 ± 0.11	0.84	0.94 ± 0.04	0.96	0.81 ± 0.10	0.84
PLS-DA	Germinated	-	54(56)	-	55(56)	82(86)	50(56)
	Non-germinated	-	11(23)	-	13(23)	23(35)	15(23)
	Accuracy	0.83 ± 0.12	**0.82**	0.87 ± 0.04	0.86	0.80 ± 0.11	0.82
	Sensitivity	0.59 ± 0.26	0.48	0.57 ± 0.13	0.61	0.57 ± 0.19	0.65
	Specificity	0.93 ± 0.08	0.96	0.98 ± 0.02	0.96	0.90 ± 0.07	0.89
RF	Germinated	-	54(56)	-	54(56)	-	53(56)
	Non-germinated	-	7(23)	-	14(23)	-	14(23)
	Accuracy	0.73 ± 0.13	0.77	0.85 ± 0.09	0.86	0.84 ± 0.09	**0.85**
	Sensitivity	0.30 ± 0.23	0.30	0.57 ± 0.19	0.61	0.53 ± 0.14	0.61
	Specificity	0.93 ± 0.08	0.96	0.97 ± 0.03	0.96	0.97 ± 0.03	0.94
NB	Germinated	-	44(56)	-	49(56)	-	46(56)
	Non-germinated	-	11(23)	-	17(23)	-	13(23)
	Accuracy	0.65 ± 0.14	0.69	0.83 ± 0.06	0.84	0.73 ± 0.14	0.74
	Sensitivity	0.57 ± 0.17	0.48	0.60 ± 0.10	0.74	0.66 ± 0.10	0.57
	Specificity	0.69 ± 0.17	0.78	0.93 ± 0.06	0.87	0.75 ± 0.15	0.82
SVM-*r*	Germinated	-	52(56)	-	55(56)	86(86)	53(56)
	Non-germinated	-	11(23)	-	14(23)	24(35)	11(23)
	Accuracy	0.78 ± 0.11	0.79	0.84 ± 0.06	0.86	0.79 ± 0.11	0.81
	Sensitivity	0.38 ± 0.27	0.48	0.58 ± 0.09	0.61	0.51 ± 0.23	0.48
	Specificity	0.93 ± 0.04	0.93	0.95 ± 0.04	0.96	0.92 ± 0.06	0.97

Note: LDA—linear discriminant analysis; PLS-DA—partial least squares discriminant analysis; RF—random forest; NB—naive Bayes; and SVM-*r*—support vector machine with radial basis kernel.

**Table 3:** Number of seeds correctly classified for different vigor classes using descriptors generated by Fourier transform near-infrared (FT-NIR) spectroscopy, X-ray imaging, and the combination of FT-NIR spectroscopy and X-ray imaging.

Method	Feature	FT-NIR		X-Ray Imaging		FT-NIR + X-Ray Imaging	
		Cross-Validation	Testing	Cross-Validation	Testing	Cross-Validation	Testing
		(n = 121)	(n = 79)	(n = 121)	(n = 79)	(n = 121)	(n = 79)
		Hits (Total)		Hits (Total)		Hits (Total)	
LDA	Non-germinated	-	13(25)	-	16(25)	-	14(25)
	Rapid germination	-	29(38)	-	37(38)	-	28(38)
	Slow germination	-	6(16)	-	0(16)	-	3(16)
	Accuracy	0.52 ± 0.06	**0.61**	0.61 ± 0.11	0.67	0.50 ± 0.08	0.57
	Sensitivity	0.51 ± 0.20	0.55	0.51 ± 0.34	0.54	0.48 ± 0.21	0.49
	Specificity	0.75 ± 0.11	0.79	0.79 ± 0.18	0.79	0.74 ± 0.12	0.76
PLS-DA	Non-germinated	-	15(25)	-	16(25)	-	12(25)
	Rapid germination	-	33(38)	-	38(38)	-	31(38)
	Slow germination	-	0(16)	-	0(16)	-	3(16)
	Accuracy	0.57 ± 0.09	**0.61**	0.62 ± 0.09	**0.68**	0.58 ± 0.05	0.58
	Sensitivity	0.50 ± 0.32	0.49	0.49 ± 0.40	0.55	0.50 ± 0.27	0.49
	Specificity	0.77 ± 0.18	0.77	0.77 ± 0.25	0.8	0.78 ± 0.17	0.76
RF	Non-germinated	-	15(25)	-	15(25)	-	13(25)
	Rapid germination	-	25(38)	-	74(38)	-	35(38)
	Slow germination	-	2(16)	-	0(16)	-	1(16)
	Accuracy	0.54 ± 0.12	0.53	0.59 ± 0.05	0.66	0.59 ± 0.10	**0.62**
	Sensitivity	0.46 ± 0.29	0.46	0.49 ± 0. 40	0.52	0.51 ± 0.34	0.50
	Specificity	0.74 ± 0.23	0.73	0.76 ± 0. 26	0.78	0.77 ± 0.23	0.77
NB	Non-germinated	-	12(25)	-	15(25)	-	13(25)
	Rapid germination	-	15(38)	-	30(38)	-	17(38)
	Slow germination	-	7(16)	-	1(16)	-	8(16)
	Accuracy	0.46 ± 0.12	0.43	0.56 ± 0.06	0.58	0.45 ± 0.12	0.48
	Sensitivity	0.49 ± 0.16	0.44	0.48± 0. 32	0.48	0.49 ± 0.18	0.49
	Specificity	0.74 ± 0.13	0.72	0.77 ± 0. 16	0.76	0.74 ± 0.11	0.75
SVM-*r*	Non-germinated	-	12(25)	-	16(25)	-	13(25)
	Rapid germination	-	28(38)	-	36(38)	-	30(38)
	Slow germination	-	2(16)	-	0(16)	-	2(16)
	Accuracy	0.56 ± 0.12	0.50	0.64 ± 0.05	0.66	0.59 ± 0.07	0.57
	Sensitivity	0.50 ± 0.26	0.45	0.53 ± 0.41	0.53	0.52 ± 0.28	0.48
	Specificity	0.76 ± 0.17	0.73	0.79 ± 0.25	0.78	0.77 ± 0.19	0.75

Note: LDA—linear discriminant analysis; PLS-DA—partial least squares discriminant analysis; RF—random forest; NB—naive Bayes; and SVM-*r*—support vector machine with radial basis kernel.
